# Constructing a Bayesian neural network model for the cross-sectional classification of psychological distress among older adults: comprehensive analysis of humor styles, communication skills, and physical activity experiences

**DOI:** 10.3389/frma.2026.1882045

**Published:** 2026-08-11

**Authors:** Keishi Soga, Keita Kamijo, Takuji Kawamura, Akari Uno, Yasuyuki Taki

**Affiliations:** 1 Smart-Aging Research Center, Institute of Development, Aging and Cancer, Tohoku University, Sendai, Japan; 2 Faculty of Liberal Arts and Sciences, Chukyo University, Nagoya, Japan; 3 Department of Aging Research and Geriatric Medicine, Institute of Development, Aging and Cancer, Tohoku University, Sendai, Japan; 4 Department of Psychology, Faculty of Humanities, Saitama Gakuen University, Kawaguchi, Japan

**Keywords:** Bayesian neural network, cross-sectional classification, humor styles, older adults, psychological distress

## Abstract

**Objectives:**

This study aimed to develop a model for the cross-sectional classification of psychological distress among older adults, using a Bayesian Neural Network (BNN) to examine the relationships among humor expressions, communication skills, physical activity (PA), and social PA experiences (with friends), and their effects on psychological distress risk.

**Methods:**

A cross-sectional survey was conducted among 5,265 Japanese adults aged 65 to 89 years. The predictor variables included humor expressions, communication skills, and social PA experiences. Depression was assessed using the K6 Psychological Distress Scale (cutoff ≥ 9). A BNN with three hidden layers was constructed with SHapley Additive exPlanations (SHAP) for feature importance identification.

**Results:**

The BNN model achieved 81.1% accuracy for high-risk detection. This study revealed self-enhancing humor coping and self-control communication skills as the strongest protective factors. Additionally, past PA experiences with friends and present PA experiences alone reduced the risk for K6-assessed psychological distress.

**Conclusion:**

The BNN model identified positive humor styles, self-controlled communication abilities, past social PA, and present PA status as important predictors of contributors to the model output for classifying K6-assessed psychological distress among older adults, highlighting the potential relevance of social engagement in communication and PA.

## 1 Introduction

Older adults experience various social losses, such as the death of significant others, severe illness of related persons, relocation of close persons, and separation/divorce, which can serve as factors that trigger psychological distress, such as depression (Förster et al., 2018). According to a World Health Organization (WHO) report (WHO Mental health of older adults, 2023) approximately 14% of adults aged 60 years and over suffer from mental disorders. Additionally, approximately 27.2% of deaths by suicide worldwide are attributed to adults aged 60 years and over (WHO Mental health of older adults, 2023). Given the complexity of psychological distress risk factors in later life, it is crucial to develop comprehensive predictive models that can identify at-risk individuals before they experience significant distress in their daily lives. Therefore, this study employed machine learning techniques to develop an early psychological distress classification model for older adults, focusing on humor expression, communication skills, and physical activity (PA) patterns, including social PA experiences.

Studies examining the relationship between humor and mental health have shown that benevolent and other-focused humor are associated with the alleviation of depression and stress, whereas humor that involves attacking others, such as sarcasm, is associated with increased levels of depression (Dionigi et al., 2023). Communication skills are essential for building and maintaining good interpersonal relationships. Multifaceted communication skills such as self-control, expressiveness, decoding ability, and assertiveness are measured using the ENDCOREs scale (Fujimoto and Daibo, 2007). Recent evidence supports this relationship, demonstrating that competence in interpersonal communication is negatively associated with depression (Çikrikçi, 2024). Specifically, individuals with deficient interpersonal communication skills may experience difficulties satisfying their basic psychological needs for autonomy, competence, and relatedness, which subsequently increase their vulnerability to anxiety, stress, and depressive symptoms.

Furthermore, intervention research has demonstrated that family-oriented communication skills training programs can significantly reduce depression, anxiety, and stress scores among older adults, with effects sustained at one-month follow-up (Ghazavi et al., 2016). Based on these findings, it can be inferred that higher levels of positive humor styles and communication skills may be associated with lower psychological distress by enabling individuals to navigate social interactions effectively, maintain meaningful relationships, and fulfill their fundamental psychological needs.

Additionally, PA plays a fundamental role in preventing psychological distress. Prospective evidence shows that PAs are key modifiers of late-life depression risk. A meta-analysis reported that mentally passive sedentary time (e.g., watching television) increases incident depression by approximately 10 % (Huang et al., 2020). Accelerometer data from 60,235 UK Biobank participants indicated that replacing 60 min/ day of sedentary behavior with moderate-to-vigorous PA lowers depressive-symptom scores by 12.5 % and reduces the odds of probable depression to 0.75 (Kandola et al., 2021), and a device-based systematic review of 51 studies (1.3 million individuals) found that the most active adults have 9 % lower odds of depression than the least active, whereas exposure to high levels of sedentary behavior confers a 9 % greater risk for depression (Wang et al., 2025). Research has shown that habitual PA improves social-cognitive functions that facilitate interpersonal relationships (Ludyga et al., 2022). Based on previous findings, it is implied that such PA-induced improvements in social cognition may confer protective effects for older adults.

Our previous study established a relationship between social PA (e.g., past and present PA experiences with friends) and humor expression, demonstrating that engagement in such activities is associated with enhanced positive humor (Soga et al., 2026). Although our previous study examined the association between PA, humor, and loneliness among older adults, this study extends this line of inquiry by focusing on psychological distress and developing a BNN-based model for its cross-sectional classification. This perspective represents an important advancement in understanding the mental health of older adults, thereby enabling the early identification of individuals who may benefit from support before experiencing significant distress in their daily lives. Consistent with our previous findings on the importance of social PA (Soga et al., 2026), this study integrated social PA experiences into a machine learning framework. This study aimed to capture the multifaceted nature of the risk factors for psychological distress.

Social interaction factors such as humor and communication skills, together with PA that supports social-cognitive processes, are thought to influence psychological distress among older adults. Nevertheless, their combined predictive value and relative importance in the risk assessment for psychological distress remain unclear. To address this gap, this study analyzed a large-scale database comprising more than 5,000 older adults using a Bayesian Neural Network (BNN), which assigns probability distributions to the network's weight parameters, and thus, captures epistemic uncertainty while estimating model parameters (Hüllermeier and Waegeman, 2021). This approach enabled a comprehensive evaluation of how these diverse psychosocial and behavioral factors collectively contribute to the classification of psychological distress. The resulting insights are expected to advance our understanding of depression-related factors in later life and provide valuable directions for future longitudinal research.

To develop a comprehensive predictive framework for the early detection of psychological distress risk in daily living contexts, this study employed a BNN to construct a detection model incorporating multiple domains: humor styles, humor coping styles, communication skills, PA patterns, and sedentary behavior. Based on our previous research findings, the model includes both social PA experiences (with friends) and solitary PA experiences across temporal dimensions (past and present). This represents the first comprehensive attempt to simultaneously integrate humor, communication, and social PA factors within a machine learning framework to predict psychological distress among older adults. We hypothesized that other-oriented factors, including humor styles that consider others, effective communication skills, and engagement in PA with others, would be associated with the reduced risk for psychological distress among older adults. This study also adopted an exploratory approach to determine the specific factors within and across these domains that emerge as the strongest predictors, as prior research has not established the relative importance of these diverse psychological and behavioral variables in psychological distress classification models. Through this analytical model approach, this study is expected to contribute to understanding the complex relationship among factors associated with the risk for psychological distress among older adults.

## 2 Method

### 2.1 Participants and procedure

This study analyzed data from the same web-based cross-sectional survey described in our previous study (Soga et al., 2026), which was conducted in November 2024. The original eligibility criteria were men and women aged 65 to 99 years who understood the study procedures and provided informed consent. The exclusion criterion was inability to provide informed consent. Because the study was designed as an exploratory, large-scale web-based survey, no additional exclusion criteria were applied based on psychological distress, comorbidities, or other clinical conditions. The study used Google Forms to target adults aged 65 to 89 years who were members of Cross Marketing Inc., which is an online survey company in Japan with a panel of over 10 million registered monitors (Cross Marketing Inc.). Therefore, individuals aged 90 years or older were not included in the present analytical sample. Sample size management and participant recruitment were administered by Cross Marketing Inc. to ensure adequate enrollment while maintaining quality standards. The participant selection process is illustrated in Supplemental Figure 1. All procedures were conducted in accordance with the Declaration of Helsinki and the Ethical Guidelines for Life Sciences and Medical Research Involving Human Subjects. Participants were provided with reward points, read explanations regarding the research procedures, and were directed to the response page after providing consent to participate in the study. Of the 5,273 participants aged 65 to 89 years who met the inclusion criteria, eight individuals who did not specify their gender were excluded, resulting in a final analytical sample of 5,265 participants (women: 2,630; men: 2,635). This study was approved by the Ethics Committee of the Graduate School of Medicine, Tohoku University (2024-1-545).

### 2.2 Measures

#### 2.2.1 K6-assessed psychological distress

Psychological distress was assessed using the Japanese version of the Kessler Psychological Distress Scale (K6: (Furukawa et al., 2008)). This scale consists of six items measuring psychological distress over the past 30 days. Each item is rated on a 5-point scale (0 = none of the time to 4 = all of the time), with higher total scores indicating more severe depressive symptoms. In this study, a cutoff score of 9 points was used, with scores of 9 or higher indicating risk for psychological distress. Participants were dichotomized into the “low-risk group” (0) for K6 total scores below 9 points and the “high-risk group” (1) for scores of 9 points or higher. We used a cutoff score of 9 or higher on the K6 scale to identify individuals with elevated psychological distress. We chose this sensitive threshold to prioritize screening sensitivity while recognizing that optimal cutoffs may vary depending on the study purpose and population characteristics, with reference to (Furukawa et al., 2008), who demonstrated high stratum-specific likelihood ratios for higher score ranges in their Japanese validation study.

#### 2.2.2 Humor styles

Humor styles were assessed using the Japanese version of the Humor Styles Questionnaire (HSQ; (Yoshida, 2012), which measures four humor styles: affiliative, self-enhancing, aggressive, and self-defeating humor through 32 items (8 items per subscale). The original 7-point scale was modified into a 4-point scale (1 = does not apply to me at all; 4 = applies to me completely) to reduce the response burden on older participants. Higher scores indicate a greater propensity toward each humor style.

### 2.3 Humor coping style

Humor coping styles were assessed using the Humor Coping Questionnaire (HCQ; (Hongo, 2019)), a 24-item scale that measures four dimensions: self-enhancing (maintaining a humorous perspective during stress), cooperative (using humor to create harmony in social situations), aggressive (using humor to attack or gain superiority), and self-joking humor coping (making fun of one's failures or shortcomings). The original 6-point response scale was modified to a 4-point scale (1 = does not apply to me at all; 4 = applies to me completely) to reduce the response burden. Each subscale contains six items, with total scores ranging from 6 to 24, where higher scores indicate greater use of a humor coping style.

### 2.4 Communication skills

Communication skills were assessed using the ENDCOREs scale (Fujimoto and Daibo, 2007). The ENDCOREs consists of 24 items across six subscales: “self-control,” “expressiveness,” “decoding,” “assertiveness,” “other-acceptance,” and “relationship adjustment.” To reduce the response burden on older participants, the original 7-point scale (ranging from quite poor to quite good) was modified to a 4-point scale (1 = poor, 2 = somewhat poor, 3 = somewhat good, and 4 = good). Higher total scores on each subscale indicated better skills in that domain. According to (Fujimoto and Daibo, 2007), the six factors of the ENDCOREs scale reflect the hierarchical structure of communication skills, which are classified into basic (self-control, expressiveness, and decoding) and interpersonal skills (assertiveness, other-acceptance, and relationship adjustment). This hierarchical structure enables a detailed examination of the multifaceted influences of communication skills on K6-assessed psychological distress among older adults.

### 2.5 Physical activity and sedentary time

Physical activity (PA) and sedentary time were assessed using the Global Physical Activity Questionnaire (GPAQ: Bull et al., 2009). PA was analyzed by converting it into metabolic equivalents (METs) per week, and sedentary behavior was measured as the total daily duration of sitting and reclining activities, reported in hours and minutes per day. For PA experiences with others, participants were asked about past and current PA experiences with others through the question, “Have you engaged in exercise or sports together with companions?” with three response options: “yes,” “no,” and “I do not know.” Additionally, for PA experiences alone, participants were asked about past and current solo exercise through the question, “Have you engaged in exercise or sports alone?” with the same three response options: “yes,” “no,” and “I do not know.”

### 2.6 Data analysis

This study employed BNNs to classify K6-assessed psychological distress among older adults and identify variables contributing to the classification. The neural network was constructed using Python (version 3.12.0). All variables included in the analysis were complete, and no missing-value imputation was required.

### 2.7 Data preprocessing and feature engineering

In the selection and preprocessing of explanatory variables, 29 variables related to participants' basic attributes, PA levels, and social skills were selected as explanatory variables. The numerical variables included 25 measurements across several domains. The basic attributes included age, exercise initiation age, and exercise duration. PA levels were assessed using work-related vigorous- and moderate-intensity METs, transport-related METs, leisure activity vigorous- and moderate-intensity METs, total METs, and sedentary time. Humor-related variables included four humor styles (affiliative, self-defeating, self-enhancing, and aggressive) and four humor-coping strategies (self-enhancing, collaborative, aggressive, and self-defeating), with total scores calculated for each. Social skills were measured across six dimensions: self-control, expressiveness, decoding, assertiveness, other-acceptance, and relationship adjustment. Total scores were computed for each subscale. Categorical variables included four measures: gender and PA experience assessed for both past and present timeframes, specifically, past PA experience with friends, past PA experience alone, present PA experience with friends, and present PA experience alone.

### 2.8 Data standardization

Numerical variables were normalized to a 0–1 range using a Min-Max Scaler. This prevented bias in the influence between variables with different scales or units and improved the learning efficiency of the neural network. Categorical variables were already encoded as 0 and 1; therefore, no additional transformations were performed. The final preprocessed dataset was constructed as a 29-dimensional feature vector by horizontally concatenating standardized numerical and categorical features. As for data splitting, to appropriately evaluate the model's generalization performance, the preprocessed dataset was split into training (70%) and test data (30%) using stratified sampling. To ensure the reproducibility of the split, a random seed (random state = 42) was used. This split allowed model training on the training data and the evaluation of the model performance on unknown data using the test data. In the model architecture, a BNN was constructed using the JAX and NumPyro libraries. The model was designed as a multilayer perceptron (MLP) consisting of three hidden layers and one output layer. Each hidden layer contained 32 units and Leaky Rectified Linear Unit (ReLU; negative slope of 0.1) was adopted as the activation function.

For prior distribution settings, normal distributions were set as priors for all weight and bias parameters. Specifically, a normal distribution with a mean of 0 and a standard deviation of 0.5 was assumed for each weight matrix, and the same normal distribution was assumed for the bias vectors. This setting appropriately modeled the parameter uncertainty and prevented overfitting. Regarding the likelihood function, the probability mass function of the Bernoulli distribution was defined as the likelihood using logit values obtained through a linear transformation in the output layer. This enabled Bayesian inference to be suitable for binary classification problems. For Markov chain Monte Carlo (MCMC) inference, Bayesian inference was conducted using the No-U-Turn Sampler (NUTS), an efficient variant of the Hamiltonian Monte Carlo method that enables stable sampling, even in high-dimensional parameter spaces. MCMC sampling was performed with a target acceptance probability of 0.9 to improve sampling stability; a maximum tree depth of 12, to expand the search range; 1,500 warm-up samples to ensure convergence; and 3,000 posterior samples, to improve estimation accuracy. Four parallel chains were used for computational efficiency. These hyperparameter settings enabled the efficient acquisition of representative samples from the posterior distribution and appropriate quantification of parameter uncertainty.

### 2.9 Cross-sectional classification and evaluation

For model classification and evaluation, predictive distributions were generated using the Predictive function, with parameters sampled from the posterior distribution. For each test sample, the final prediction probability was calculated as the mean of the prediction probabilities across all posterior samples. To improve model performance, the classification threshold was set to 0.3 instead of the conventional 0.5, to reduce false negatives and improve recall (sensitivity) for identifying individuals at risk of K6-assessed psychological distress. The model performance was comprehensively evaluated using accuracy, precision, recall, F1-score, and a confusion matrix.

### 2.10 Model evaluation and interpretation

For feature importance analysis, SHapley Additive exPlanations (SHAP) values were calculated to quantify the contribution of each feature to the model output. SHAP is a method based on cooperative game theory that improves model interpretability by fairly distributing the contribution of each feature to the machine learning model output. In the SHAP analysis implementation, 500 samples were randomly selected from the training data as the background dataset using Independent Masker, which was determined by considering the balance between computational efficiency and estimation accuracy. To ensure reproducibility, a fixed random seed (PRNGKey(0)) was used for all stochastic operations during SHAP value computation. SHAP values were calculated for 500 samples from the test data, with a maximum of 100 evaluations per sample. For visualization and interpretation, the SHAP value results were visualized using summary plots, which enabled a simultaneous understanding of the importance ranking of each feature, direction of contribution to the model output, and relationships with feature value distributions. This analysis identified the aspects of PA and social skills that contributed most to the classification of K6-assessed psychological distress among older adults.

## 3 Results

### 3.1 Model convergence and classification performance

**Figure 1:**
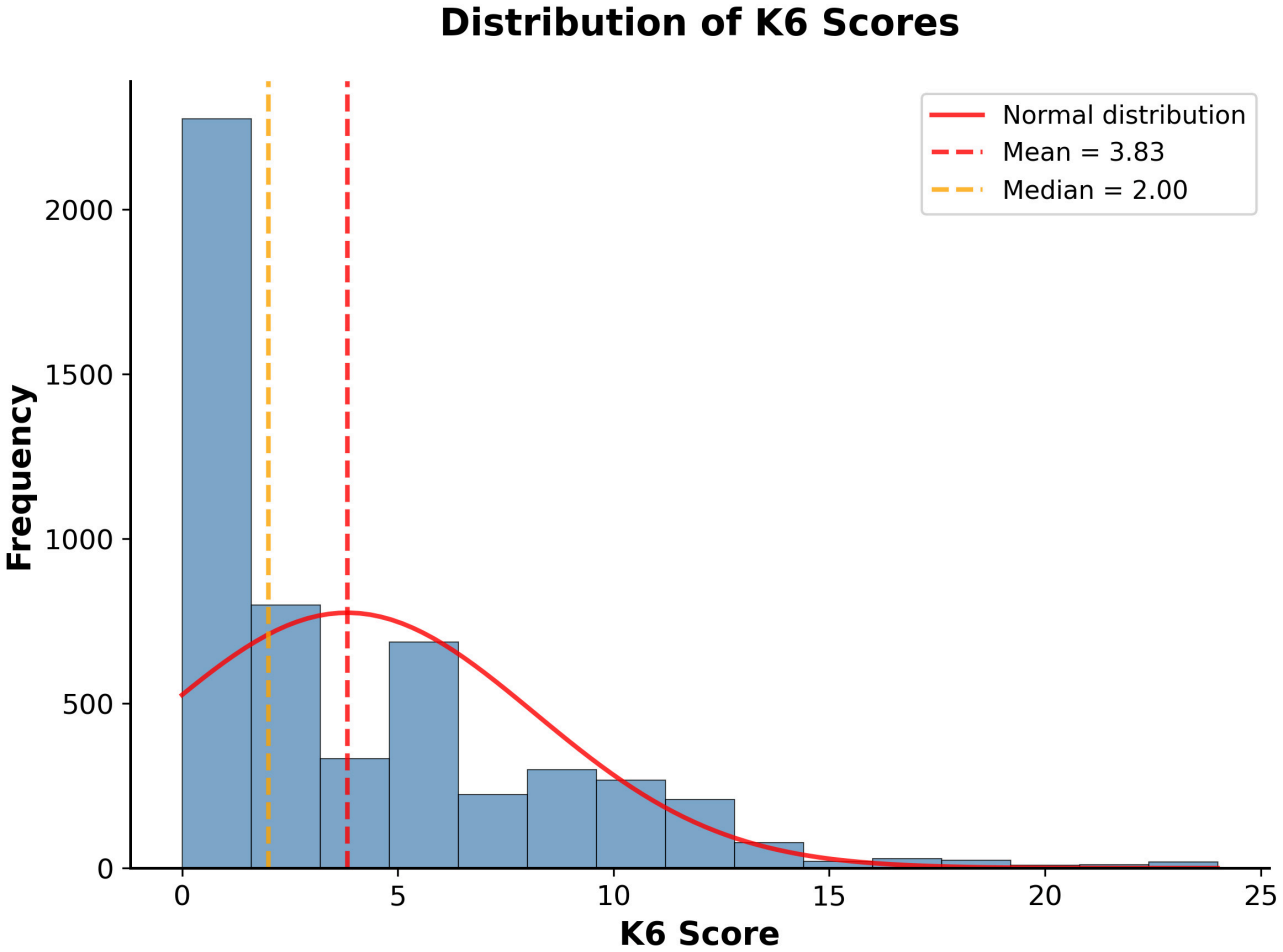
Distribution of K6 score.

Regarding the convergence of MCMC sampling by the Bayesian neural network, the R-hat statistic for all parameters was 1.0, indicating sufficient model convergence. The distribution of the K6 scores is illustrated in Figure 1.

**Figure 2:**
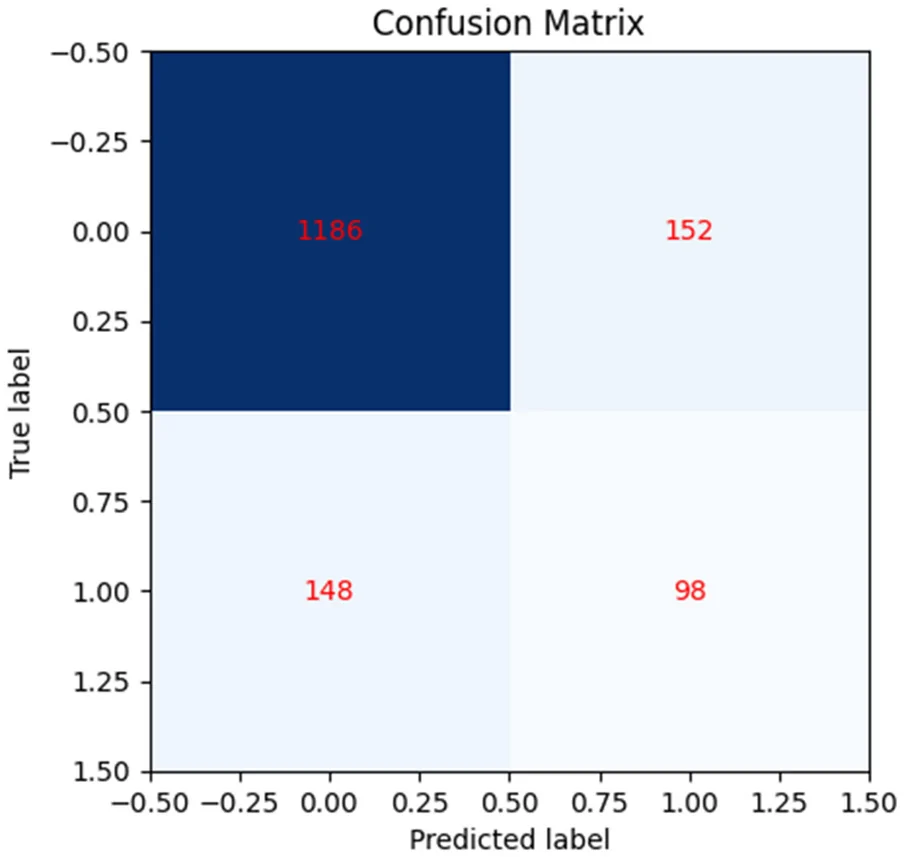
Confusion matrix for binary classification using a Bayesian neural network. Prediction probabilities were computed from posterior samples, and final classification labels were determined using a threshold of 0.3. The vertical axis represents true labels, and the horizontal axis represents predicted labels. Numbers in each cell indicate the count of corresponding samples.

In the performance evaluation of the K6-assessed psychological distress classification model for older adults, when the classification threshold was set to 0.3, accuracy was 0.811, with correct classification in approximately 81% of all cases. Regarding the detection of the high K6-assessed psychological distress risk group (K6 ≥ 9 points), precision was 0.392, recall was 0.398, and F1-score was 0.395. The results are shown in Figure 2.

Classification threshold optimization was implemented to improve classification performance. F1-scores were calculated and compared for 50 different threshold settings ranging from 0.15 to 0.3. The optimal classification threshold was determined to be 0.196. By lowering the threshold from 0.3 to 0.196, the F1-score improved by 13% (0.395 to 0.447), with performance at the optimal threshold of 0.196 achieving accuracy of 0.753, precision of 0.343, recall of 0.642, and F1-score of 0.447. Particularly noteworthy was the substantial improvement in recall from 0.398 to 0.642 (61.3% improvement), indicating that approximately 64% of actual high-risk individuals could be correctly detected. However, precision decreased slightly from 0.392 to 0.343, and the accuracy declined from 0.811 to 0.753 (Supplemental Figures 2 and 3). In addition, the predicted probabilities and their corresponding 95% credible intervals for individual test samples are presented in Supplementary Figure 4.

Visualization of Feature Contribution Using SHAP Values

**Figure 3:**
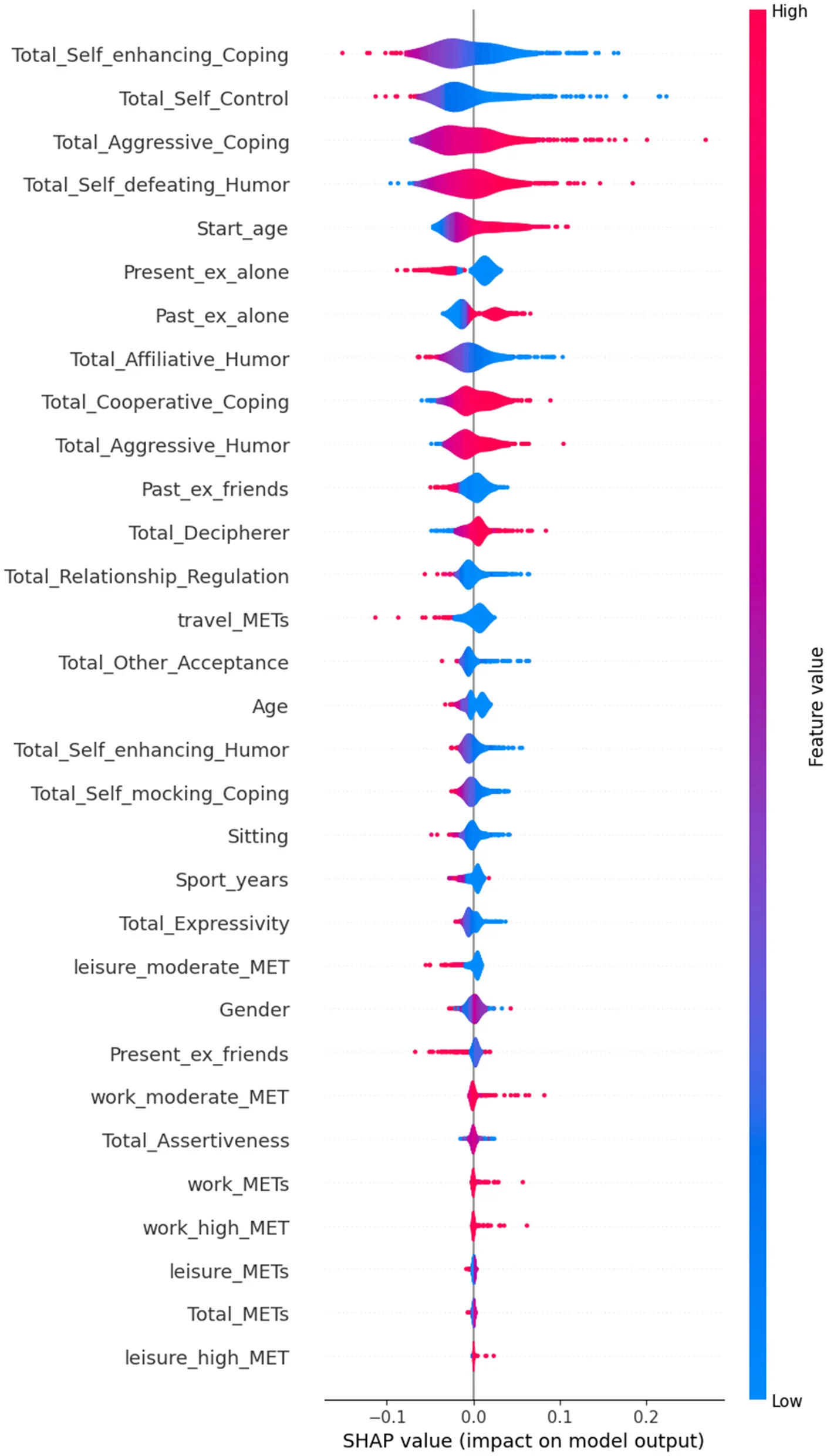
Model interpretability analysis using SHAP (SHapley Additive exPlanations) values. The plot displays the marginal contribution of each input feature to the posterior predictive probability, computed using Shapley values for model-agnostic explanation. Feature importance is ranked by the magnitude of SHAP values across 500 test instances, with positive values indicating increased prediction probability.

Figure 3 shows the contribution of each feature to the classification of K6-assessed psychological distress among older adults using SHAP values. This analysis shows how the value of each feature (represented by color) influenced the classification results (SHAP value on the horizontal axis). Positive SHAP values indicated contribution to an increased risk for K6-assessed psychological distress, whereas negative values indicated contribution to a decreased risk for K6-assessed psychological distress. Among all features, mean absolute SHAP values of self-enhancing humor coping (high values reduce the risk for K6-assessed psychological distress), self-control (high values reduce the risk for K6-assessed psychological distress), aggressive humor coping (high values increase the risk for K6-assessed psychological distress), self-defeating humor (high values increase the risk for K6-assessed psychological distress), age at which exercise was initiated (older age at exercise initiation increases the risk for K6-assessed psychological distress), present PA experience alone (experience reduce the risk for K6-assessed psychological distress), past PA experience alone (experience increases the risk for K6-assessed psychological distress), affiliative humor (high values reduce the risk for K6-assessed psychological distress), cooperative humor coping (high values increase the risk for K6-assessed psychological distress), aggressive humor (high values increase the risk for K6-assessed psychological distress), past PA experience with friends (experience reduces the risk for K6-assessed psychological distress) were identified. Of these features, six were related to humor styles and communication skills, and three were related to past and present PA experiences.

## 4 Discussion

This study aimed to develop a model for the cross-sectional classification of K6-assessed psychological distress among older adults using BNNs, examining the multifaceted influences of humor styles, humor coping styles, communication skills, PA and sedentary behavior, and social PA experiences on the risk for K6-assessed psychological distress. Our findings revealed that the BNN model achieved relatively high overall accuracy (0.811) when using a classification threshold of 0.3. Through classification threshold optimization, the model achieved improved classification performance with an F1-score of 0.447 and recall of 0.642, enabling the detection of approximately 64% of actual high-risk individuals, which is particularly valuable for early intervention and prevention efforts in community settings.

As shown in Figure 3, the SHAP analysis indicated that social skills, such as self-control and humor-related factors, including self-enhancing and affiliative humor coping styles, were important factors contributing to the classification of K6-assessed psychological distress. Furthermore, past PA experience with friends and present PA experience alone were found to reduce the risk for K6-assessed psychological distress. Conversely, humor styles such as self-defeating, cooperative, and aggressive humor coping styles, as well as past PA experience alone were found to increase the risk for K6-assessed psychological distress. These findings suggest that past PA experience with others and present PA experience alone, combined with the appropriate use of humor styles and the development of social skills, are important factors in reducing the risk for K6-assessed psychological distress among older adults.

Our study advances machine learning approaches to predict K6-assessed psychological distress by addressing the key limitations of existing research. Recent studies have demonstrated promising performance using clinical biomarkers and demographic variables. Nemesure et al. (2021) achieved AUC values of 0.67 for depression prediction using electronic health records. Vu et al. (2025) reported an F1-score of 0.69 using XGBoost with the NHANES dataset, focusing on socioeconomic and health-related factors. However, these approaches typically rely on variables that offer limited intervention targets. Our BNN approach achieved comparable performance (accuracy: 0.753, Precision: 0.343, and recall: 0.642, F1-score: 0.447) while uniquely incorporating social psychosocial factors, including humor styles, communication skills, and social PA experiences. By quantifying the relative importance of these modifiable factors through SHAP analysis, our study provides insight into factors relevant to the classification of K6-assessed psychological distress and may inform the development of future intervention strategies for older adults.

Our BNN analysis with SHAP feature importance revealed several novel insights that advance our understanding of the cross-sectional classification of K6-assessed psychological distress among older adults. Most notably, to the best of our knowledge, this study is the first to demonstrate that self-enhancing humor coping and affiliative humor styles are associated with reduced risk of K6-assessed psychological distress among all humor-related variables examined. This identification of specific protective humor types was only detectable using our neural network learning approach, which captured complex non-linear interactions between multiple psychological and behavioral factors. Traditional statistical methods examining single variables or linear relationships would likely have overlooked the hierarchical importance of these humor dimensions, highlighting the value of advanced computational approaches in mental health research.

Although our cross-sectional design could not establish causal relationships, previous intervention research has provided valuable insights that have improved our predictive model. A randomized controlled study indicated that 8 weeks of training, including sessions focused on identifying personal humor preferences, cultivating playfulness, practicing laughter, and using humor to cope with stress, significantly improved the overall sense of humor in the intervention group (Momtaz et al., 2020). The enhancement of not only individuals' sense of humor but also their life satisfaction was demonstrated by the Seven Humor Habits Program, which included cultivating playfulness, enhancing laughter frequency, developing verbal humor skills, finding daily humor, self-directed humor, and stress-related humor coping (Ruch et al., 2018).

Furthermore, Wellenzohn et al. (2018) found significant correlations between sense of humor and both happiness and depressive symptoms, and demonstrated that changes in sense of humor following intervention served as predictive factors for subsequent changes in happiness and depressive symptoms. These findings support the notion that humor is a learnable skill, rather than a fixed trait. Importantly, our BNN model's identification of self-enhancing humor coping and affiliative humor as the most powerful protective factors provides valuable evidence for understanding the humor dimensions that are beneficial in reducing K6-assessed psychological distress among older adults. Future humor enhancement programs specifically targeting these protective humor styles could potentially improve both the accuracy of K6-assessed psychological distress classification models and the effectiveness of preventive strategies among older adults.

Among the communication skills, our SHAP analysis identified self-control as the most important contributor to the model output, surpassing other competencies in terms of the importance of K6-assessed psychological distress classification. This finding extends beyond previous correlational studies by demonstrating that, when considered alongside humor styles and PA experiences, emotional self-regulation emerges as a critical communication competency for mental health among older adults. Self-control encompasses the ability to effectively manage emotions and behaviors in social interactions, and high self-control levels are associated with more effective conflict management and reduced avoidant behaviors (Bornstein et al., 2017). Our BNN model revealed that other communication competencies—such as expressiveness, decoding ability, assertiveness, other-acceptance, and relationship adjustment—were not among the most influential predictors, indicating that emotional self-control may be more critical for psychological well-being than assertive communication or active self-expression.

Our integrated analysis revealed that past PA experience with friends and present PA experience alone were important variables contributing to the classification of lower K6-assessed psychological distress, whereas past PA experience alone contributed to the classification of higher K6-assessed psychological distress. These findings challenge the conventional assumption that any type of PA is uniformly beneficial for the mental health of older adults and further suggest that both the social context and the timing of PA experience are critical determinants of psychological outcomes. The contrasting effects of past and present solitary PA warrant particular attention. Given that a lack of social interaction increases the risk for depression (Zhu et al., 2024), our study suggests that past PA experience alone among older adults may reflect underlying social isolation patterns (such as living alone or lack of social connections) rather than PA itself being inherently harmful, indicating that past PA patterns may serve as an indicator of broader social vulnerability that increases the risk for psychological distress. However, a substantial body of evidence consistently demonstrates that PA itself exerts beneficial effects on mental health, regardless of its social context (Singh et al., 2023). Therefore, present PA experience alone may retain its protective effects through psychological mechanisms inherent to physical activity itself, such as improvements in mood and stress regulation. In this light, current solitary PA may reflect positive health behaviors rather than social isolation.

The capacity of the BNN model to simultaneously consider social and individual PA patterns revealed that the social context of PA may be more important than the activity itself for preventing psychological distress. The observation that PA experiences with friends contributed to a lower risk for K6-assessed psychological distress suggests that social interaction during PA provides psychological benefits beyond mere physiological effects. Our findings align with and extend previous mechanistic research demonstrating that PA improves social cognition through neural changes in autonomic nervous system functions, central nervous system networks, and oxytocin-mediated processes (Ludyga et al., 2022). This interpretation is supported by evidence showing that group exercise has greater antidepressant effects than individual exercise (Li et al., 2024). Furthermore, systematic reviews have demonstrated that humor-infused interventions such as laughter yoga, which combines PA with social laughter and positive emotions, significantly improve mental health outcomes, including depression, loneliness, and quality of life, among older adults (Kuru Alici and Arikan Dönmez, 2020).

Self-enhancing humor coping and self-control communication skills, along with social PA experiences, suggest that these factors may share common neural substrates related to social cognition and emotion regulation. Research indicates that these behavioral domains involve overlapping neurobiological systems. Humor processing engages the medial prefrontal cortex, with subjective funniness ratings showing positive correlations with medial prefrontal cortex activity (Iidaka, 2017). Social cognition relies on the engagement of prefrontal areas (i.e., the anterior cingulate gyrus) involved in processing other-oriented information (Apps et al., 2016), and PA, especially when it involves social interaction, may support prefrontal cortical networks relevant to social cognition (Ludyga et al., 2022). These shared neurobiological substrates may underlie the relationships observed in our integrated analysis.

Our previous study provided supporting evidence for these interconnections, demonstrating that social PA is significantly associated with the expression of positive humor among older adults (Soga et al., 2026). Individuals who demonstrate positive humor styles are also likely to possess improved self-control communication abilities and engage in socially connected PA. This pattern suggests potential bidirectional relationships among these factors. Effective communication skills may facilitate the development of positive humor styles and successful social PA partnerships, whereas shared PA may provide contexts for practicing humor and communication abilities, thereby creating a mutually reinforcing cycle of social competence and wellbeing.

Although we successfully developed a BNN-based model for the cross-sectional classification of K6-assessed psychological distress, several limitations should be acknowledged. First, because the study used a cross-sectional design, causal relationships between the variables could not be established. The relationships between PA and humor abilities and social skills may extend beyond the direct antidepressant effects of PA. Although PA is well established to be beneficial for the prevention of depression, our findings suggest that the social dimensions of PA may provide additional pathways for mental health benefits through the enhancement of humor and communication competencies. However, determining whether PA facilitates the development of humor and social skills or whether these relationships reflect other underlying factors requires carefully designed intervention studies that can establish causal pathways among these interconnected behavioral domains. SHAP analysis demonstrated the relative contribution of each feature within the model and did not provide evidence of causality. Second, reliance on self-reported questionnaires may introduce social desirability bias and may not provide an objective assessment of communication skills and humor abilities. It is important to acknowledge that our study focused on classifying self-reported depressive symptoms rather than clinically diagnosed depression, and substantial improvements in classification performance and external validation are required before the model can be considered for clinical use. Although the K6 is a validated tool for identifying individuals at risk for mental health problems (Furukawa et al., 2008), our findings cannot be directly extrapolated to clinical depression diagnoses. Future studies should validate these findings through structured clinical interviews and other diagnostic assessments. As highlighted by Squires et al. (2023), depression detection systems that rely on self-reported data exhibit significant performance limitations. Their review emphasized that existing methods fail to capture the inherent uncertainty within self-reported mental health data, recommending a BNN to compensate for this limitation. Although our BNN approach achieved a performance comparable to that of recent studies (F1-score: 0.447, recall: 0.642), Squires et al. (2023) highlighted that no machine learning-based studies in the field of psychiatry have yielded demonstrable improvements in patient outcomes. Future research should focus on enhancing model performance through clinical validation, larger and more diverse datasets, and potentially incorporating multimodal data sources to achieve clinically meaningful prediction accuracy that can support real-world intervention strategies. Third, this study was conducted exclusively in Japan, which may limit the generalizability of the findings to older adults from different cultural contexts or countries. Additionally, the absence of PA experience with friends as a predictor of K6-assessed psychological distress should be interpreted with caution. In this study, only approximately 16% of participants reported engaging in current PA with friends. This limited sample size for the subgroup may have resulted in insufficient statistical power to detect potential protective effects. Therefore, the possibility that socially engaged PA contributes to mental health cannot be entirely ruled out, and further studies with a more balanced distribution of exercise patterns are needed to clarify these relationships.

Furthermore, the present study did not include an independent external validation dataset. Therefore, the reported model performance should be interpreted as internal performance within the present dataset and may not be generalizable to other populations or settings. Future studies should validate the model using independent cohorts from different populations, regions, and data collection settings to evaluate its generalizability and practical applicability. Several established predictors of K6-assessed psychological distress, including socioeconomic status, chronic diseases, neurological disorders, cognitive impairment, medication use, social support, marital status, previous psychiatric history, and a history of physical trauma, were not available in the present dataset and therefore could not be incorporated into the model. Participants with these conditions could not be excluded or adjusted for in the analysis. As a result, unmeasured clinical, socioeconomic, and psychosocial factors may have influenced the model performance and the interpretation of the associated factors. Future studies should develop and validate models that incorporate detailed clinical, socioeconomic, and psychosocial information. In addition, the present study did not benchmark the BNN model against conventional statistical or machine-learning models. Therefore, we cannot determine whether the additional complexity of the BNN provided meaningful improvement over simpler approaches. Future studies should directly compare the BNN with conventional classification models to evaluate differences in performance, calibration, and interpretability. Finally, model calibration was not formally assessed; therefore, the reliability of the predicted probabilities remains uncertain. Future studies should evaluate calibration using appropriate metrics and independent validation datasets.

In conclusion, to the best of our knowledge, this study represents the first comprehensive investigation to examine diverse factors related to K6-assessed psychological distress among older adults using a BNN, which provided unique advantages in modeling complex, non-linear relationships among multiple variables while incorporating uncertainty quantification in classification probabilities. Most notably, our integrated analytical approach revealed several novel insights that would be difficult to detect using traditional statistical methods, including the identification of positive humor styles and self-control communication skills, which are associated with reduced risk of K6-assessed psychological distress. Furthermore, whereas past PA experience alone may serve as an indicator of social vulnerability, present PA experience alone may reflect autonomous health behaviors with protective potential. These findings suggest that positive humor styles, self-control communication abilities, socially engaged PA, and the encouragement of present PA experience may be important targets for future research on approaches for preventing psychological distress among older adults. In addition, past solitary PA patterns may serve as early warning indicators for identifying at-risk individuals. Our predictive framework provides valuable insights for understanding the complex relationships of psychosocial factors associated with the risk for K6-assessed psychological distress, and the early identification of individuals exhibiting these newly identified risk patterns is crucial for supporting mental health in aging populations. Future research should prioritize longitudinal and intervention studies targeting these factors to establish causal relationships and develop evidence-based approaches informed by our comprehensive predictive neural network model.

## Data Availability

The analysis scripts are publicly available and can be accessed at https://github.com/KeishiSoga/BNN_Psychological_Distress_predictivemodel. The raw data are available from the corresponding author upon reasonable request.
