# Supplementary material for: Constructing a Bayesian neural network model for the cross-sectional classification of psychological distress among older adults: comprehensive analysis of humor styles, communication skills, and physical activity experiences
Source: Front Res Metr Anal. 2026 Aug 11;11:1882045. doi: 10.3389/frma.2026.1882045 (PMC13503313; doi:10.3389/frma.2026.1882045)
Supplement: Supplementary file 1 [file Data_Sheet_1.docx]

Supplemental Figure 1. The flow of participant selection.

Supplemental Figure 2. Relationship between classification threshold and F1-score. F1-scores were evaluated on test data using 50 different threshold values ranging from 0.15 to 0.3 applied to prediction probabilities obtained from the Bayesian neural network. The optimal threshold was 0.196 with an F1-score of 0.447.

Supplemental Figure 3. Confusion matrix using optimized classification threshold. The Bayesian neural network predictions were classified using the F1-score maximizing threshold of 0.196. The vertical axis represents true labels and the horizontal axis represents predicted labels, with numbers in each cell indicating sample counts.

Supplemental Figure 4. Predictive probabilities and 95% credible intervals for the test samples. Blue points represent the posterior mean probability of elevated K6-assessed psychological distress for each test sample, and error bars indicate the corresponding 95% credible intervals. Red points represent the observed binary outcome labels.
